# Novel Anti-Trop2 Nanobodies Disrupt Receptor Dimerization and Inhibit Tumor Cell Growth

**DOI:** 10.3390/pharmaceutics16101255

**Published:** 2024-09-27

**Authors:** Junwen Deng, Zhongmin Geng, Linli Luan, Dingwen Jiang, Jian Lu, Hanzhong Zhang, Bingguan Chen, Xinlin Liu, Dongming Xing

**Affiliations:** 1 The Affiliated Hospital of Qingdao University, Qingdao University, Qingdao 266071, China; 2021021179@qdu.edu.cn (J.D.); gzmin-qdu@qdu.edu.cn (Z.G.); zhz17805982993@163.com (H.Z.); 2 Qingdao Cancer Institute, Qingdao 266071, China; 3 Noventi Biopharmaceuticals Co., Ltd., Shanghai 201203, China; ll0527ll@163.com (L.L.); jiangdingwen@auambio.com (D.J.); luj@noventi.com.cn (J.L.); chenbg@noventi.com.cn (B.C.); 4 School of Life Sciences, Tsinghua University, Beijing 100084, China

**Keywords:** anti-Trop2 nanobody, epitopes, anti-tumor efficacy

## Abstract

**Background:** Trop2 (trophoblast cell-surface antigen 2) is overexpressed in multiple malignancies and is closely associated with poor prognosis, thus positioning it as a promising target for pan-cancer therapies. Despite the approval of Trop2-targeted antibody–drug conjugates (ADCs), challenges such as side effects, drug resistance, and limited efficacy persist. Recent studies have shown that the dimeric forms of Trop2 are crucial for its oncogenic functions, and the binding epitopes of existing Trop2-targeted drugs lie distant from the dimerization interface, potentially limiting their antitumor efficacy. **Method:** A well-established synthetic nanobody library was screened against Trop2-ECD. The identified nanobodies were extensively characterized, including their binding specificity and affinity, as well as their bioactivities in antigen-antibody endocytosis, cell proliferation, and the inhibition of Trop2 dimer assembly. Finally, ELISA based epitope analysis and AlphaFold 3 were employed to elucidate the binding modes of the nanobodies. **Results:** We identified two nanobodies, N14 and N152, which demonstrated high affinity and specificity for Trop2. Cell-based assays confirmed that N14 and N152 can facilitate receptor internalization and inhibit growth in Trop2-positive tumor cells. Epitope analysis uncovered that N14 and N152 are capable of binding with all three subdomains of Trop2-ECD and effectively disrupt Trop2 dimerization. Predictive modeling suggests that N14 and N152 likely target the epitopes at the interface of Trop2 *cis*-dimerization. The binding modality and mechanism of action demonstrated by N14 and N152 are unique among Trop2-targeted antibodies. **Conclusions:** we identified two novel nanobodies, N14 and N152, that specifically bind to Trop2. Importantly, these nanobodies exhibit significant anti-tumor efficacy and distinctive binding patterns, underscoring their potential as innovative Trop2-targeted therapeutics.

## 1. Introduction

Trop2, also known as trophoblast cell surface antigen 2, is a type I cell surface glycoprotein encoded by the *TACSTD2* gene. The expression of Trop2 in normal tissues is fairly restricted. It is primarily present in epithelial cells and plays a crucial role in embryonic development. In contrast, the overexpression of Trop2 is observed in various tumor cells and plays a vital role in regulating tumor cell self-renewal, proliferation, and transformation [1,2,3]. Trop2 consists of a hydrophobic leader peptide (AA 1-26), an extracellular domain (AA 27-274), a transmembrane domain (AA 275-297), and a cytoplasmic tail (AA 298-323). Trop2-ECD is further subdivided into cysteine-rich domain (CRD), thyroglobulin type-1 (TY), and cysteine-poor domain (CPD) [4]. Previous research confirmed that Trop2 is capable of regulating intracellular and intercellular signaling pathways, including but not limited to MAPK, JAK2/STAT3, and PI3K/Akt. This is achieved by executing a series of serine residue phosphorylation, intramembrane proteolysis, and the formation of membrane-localized protein complexes [5,6,7,8,9,10,11,12,13,14,15,16,17,18]. These mechanistic pathways induced by Trop2 contribute to the promotion of tumorigenesis, malignant growth, and metastasis [5]. Therefore, Trop2 has emerged as a highly attractive therapeutic target in solid tumors.

The current primary approach for addressing Trop2-positive tumors centers on ADCs. Over fifteen Trop2-targeted ADCs are undergoing clinical investigation. These include sacituzumab govitecan (SG), DS-1062, and SKB-264 etc. [19]. Significantly, SG has been approved for the treatment of patients with unresectable locally advanced or metastatic triple-negative breast cancer (mTNBC) who have received at least two systemic therapies [5]. Nevertheless, the objective response rate (ORR) of these drugs in clinical trials is typically below 40%, and frequently accompanied by treatment-related grade 3 or higher adverse events, with the most common being interstitial pneumonia and neutropenia [5,20,21,22,23,24,25,26,27,28,29]. The part factor contributing to these side effects is likely the expression of Trop2 in normal tissues, resulting in potential on-target toxicity in non-tumor tissues [30]. Furthermore, recent studies described that *Trop2* and *TOP1* gene can experience mutations, resulting in resistance to SG. The Trop2 encoded by *TACSTD2^T256R^* showed altered subcellular localization, mostly localized in the cytosol, which weakened the binding of SG [31]. In conclusion, low response rates, notable side effects, and the emergence of drug resistance remain challenging for the treatment of Trop2-positive tumors. Therefore, there is still a need for the development of novel Trop2-targeted therapeutics to maximize the clinical benefit.

The oligomeric complexity of Trop2 is considered to play a critical role in promoting tumor progression [32,33]. Crystal structures revealed that Trop2-ECD could form *cis-*dimers or *trans-*dimers [34]. Specifically, TY and CPD domains contribute to the assembly of *cis*-dimers, while the CPD domain mediates the formation of *trans*-dimers. The *cis-* and *trans-*dimers can further assemble into *cis-* and *trans-*tetrameric through a non-overlapping interface. These various oligomeric characteristics of Trop2 could induce its functions in cell-cell communications and regulate oncogenic signaling pathways. It could be an effective treating Trop2-positive tumors strategy that develops targeted drugs to block Trop2 assembly and consequently affect its oncogenic functions. Epitope analysis showed that hRS7, the antibody backbone of SG, binds to Q237-Q252 of Trop2-ECD, far from the *cis*- or *trans*-interface. This suggests that the approved agent SG does not interfere with the dimeric assembly of Trop2, which could be the reason that hRS7 alone does not effectively suppress tumors [34,35]. It is noteworthy that the majority of existing Trop2-targeting antibodies and the antibody backbone of Trop2-targeted ADCs, including T16, 162-46.2, E1, mAbMOv16, cAR47A6.4.2, 77220, MM0588, YY-01, and hRS7, predominantly target the immunodominant epitopes located within the N-terminal region of Trop2-CPD (D146-T274) [36,37,38,39]. This suggests that these antibodies and ADCs, akin to hRS7, did not block Trop2 multimerization. Additionally, the immunodominant epitope exhibits comparable accessibility in both tumor cells and normal tissues, potentially leading to a lack of cancer specificity [38,40,41,42]. Thus, the discovery of antibodies that recognize sites beyond the immunodominant epitope could potentially overcome the current limitations of Trop2-targeted therapy [43].

Nanobodies, also known as VHH domains or single domain antibodies, confer unique advantages that are not possessed by traditional antibodies [44]. For instance, nanobodies can access cryptic epitopes by penetrating clefts on the protein surface or domain-domain interfaces [45]. Given the challenge of intricate oligomerization and inaccessible epitopes, creating anti-Trop2 nanobodies could be an alternative strategy to effectively inhibit Trop2-positive tumors. A recent study highlighted that the anti-Trop2 nanobodies mediated the inhibition of tumor cell migration. However, their efficacy is limited in reducing the proliferation of Trop2-positive tumor cells and the binding epitope has not yet been elucidated [46].

Here, we obtained two anti-Trop2 nanobodies (N14 and N152) from a synthetic nanobody library. N14 and N152 induce receptor internalization and inhibit cell growth of Trop2-positive tumor cells. Epitope analysis revealed that the binding epitopes of N14 and N152 are not confined to the immunodominant epitopes previously reported. Instead, they span all three subdomains of the Trop2-ECD and are likely located at the interface of *cis*-dimerization. Moreover, this is the first report of anti-Trop2 nanobodies capable of inhibiting the dimer assembly of Trop2. These findings suggest that N14 and N152 may serve as effective therapeutic agents for Trop2-positive tumors.

## 2. Materials and Methods

### 2.1. Cell Lines and Antibodies

NCI-N87 and MDA-MB-231 were purchased from American Type Culture Collection (ATCC) and cultured at 37 °C with 5% CO_2_. The control antibodies hRS7 and C5G2 were produced in our laboratory.

### 2.2. Phage Biopanning

The synthetic nanobody library (ASyNAL) was previously constructed and its quality control and diversity were validated [47]. To initiate the panning process, Trop2-ECD-Fc (Trop2-ECD fused with an Fc tag) and Fc fragment were separately added to two wells of a 96-well microplate and incubated overnight. The solution was then discarded, and 2% milk was added to block for 1 h. After blocking, the wells were washed with PT buffer and the phage library (100 μL/well, about 3.0 × 10^12^ phage clones) was added to the Fc wells for preincubation to remove the nonspecific binders. Subsequently, the phage solution was transferred to the Trop2-ECD-Fc wells and allowed to bind for 1 h. The wells were washed again with PT buffer to remove any unbound phage. The bound phages were eluted using 100 mM HCl and neutralized with 1.0 M Tris-HCl. The resulting phage solution was then used to infect *E.coli* XL1-Blue (Stratagene, San Diego, CA, USA) for 30 min. M13KO7 helper phages (NEB, N0315S) were introduced for 45 min of superinfection at a final concentration of 1 × 10^10^ phage/mL. The solution was subsequently cultured in 2YT medium supplemented with Carb and Kana at 32 °C. Following overnight culture, the supernatant was precipitated with PEG/NaCl (20% PEG 8000/2.5 M NaCl). The precipitated phage was resuspended in 1 × PBS and used as the input phage for the next round of panning. In this study, two rounds of panning were employed to enrich the Trop2-specific phage particles.

### 2.3. Phage ELISA

The Trop2-ECD-Fc (100 ng/well) and Fc fragment (100 ng/well) were coated in wells of 96-well NUNC microplates and incubated at 4 °C overnight. Subsequently, the solution was discarded, and 2% milk was added to block nonspecific binding for 1 h. The phage solution was then added and allowed to react at room temperature for 1 h. Afterward, unbound phages were washed away using PT buffer, and 50 μL of anti-M13/HRP conjugate (Sino Biological, Beijing, China) was incubated for 30 min. Another wash with PT buffer was performed, followed by the addition of 50 μL of TMB substrate. The color development was carried out according to the manufacturer’s instructions. To stop the reaction, 1.0 M H_3_PO_4_ (100 μL/well) was added. The signal was measured at 450 nm using a BioTek plate reader, and the hTrop2-ECD-Fc/Fc ratio was calculated. Wells with a ratio greater than 5 were selected for sequencing, resulting in 10 unique sequences of anti-Trop2 nanobodies. The data were analyzed using GraphPad Prism 8.

### 2.4. Anti-Trop2 Nanobodies Expression and Purification

In alignment with methodologies utilized in previous studies [44,47]. Specifically, the DNA sequence encoding the nanobodies was cloned into the vector pET22b, and a C-terminal 6 × His tag was added using the One Step Cloning Kit (Vazyme, C112, Nanjing, China). The expression constructs were transformed into BL21 (DE3) and plated on LB medium plates containing ampicillin. When the OD_600_ value reached 0.5~0.6, 0.2 mM IPTG was added. The bacterial solution was then cultivated at 18 °C overnight to induce protein expression. The pellets were collected and treated with a lysis buffer. Following this, the lysate was heated at 60 °C for 0.5 h to remove denatured proteins. The supernatant was purified using AKTA protein purification equipment and a Ni Bestarose FF column (BestChrom, AA0051, Shanghai, China). The eluted nanobodies were exchanged with 1 × PBS buffer using Amicon Ultra4 Centrifugal Filter Units (Millipore, Burlington, MA, USA).

The nanobody DNA sequence was cloned into the pSCSTa vector followed by a C-terminal Fc tag. The plasmid was then amplified and extracted using the endotoxin-free plasmid midiprep kit from AXYGEN. To complete transient transfection, Expi 293 cells were seeded at a density of 3 × 10^6^ cells/mL and incubated overnight at 37 °C with 8% CO_2_. The following day, the medium was replaced with fresh OPM-CD05 Trans293 medium. Both the plasmid and the 40 kDa linear PEI (Polysciences, 24765-1, Warrington, PA, USA) were diluted separately using OPM-CD05 Trans293 medium. Subsequently, the diluted PEI was dropwise to the diluted plasmid, while flicking the tube to ensure thorough mixing. The mixture was incubated for 20 min at room temperature, shielded from light, to facilitate the formation of PEI-plasmid complexes. Finally, the transfection complexes were transferred to the Expi 293 cells. After 16 h of transfection, 5% feed was added to the cells, and cultivation continued for 3~4 days. The supernatant was collected, and VHH-Fc was purified using AKTA protein purification equipment and Protein A affinity chromatography with AT Protein A Diamond Plus (BestChrom, AA402305). All purified protein concentration was determined using the BCA method.

### 2.5. SDS-PAGE

For the SDS-PAGE procedure, 15% polyacrylamide gel was prepared and assembled into the gel cassette. Subsequently, a mixture comprising 5 µg of anti-Trop2 nanobody and 1 × loading buffer heating at 95 °C for duration of 10 min. After denaturation, the mixture and the marker were loaded into separate wells on the gel. The gel was run in an electrophoresis chamber with a constant voltage of 100 V applied until the proteins separated according to their molecular weights. After electrophoresis, the gel was removed from the cassette and stained with Coomassie blue for subsequent analysis.

### 2.6. Size-Exclusion Chromatography

The nanobody solution underwent filtration using a 0.2 μm filter (Merck, PLBC07610, Rahway, NJ, USA) and was subsequently subjected to HPLC analysis on a Waters ACQUITY UPLC H-Class system. The mobile phase consisted of PBS, with a gel permeation chromatography column (TSKgel G3000SWXL, Tosoh Bioscience, 0008541, Tokyo, Japan) serving as the stationary phase. Before sample injection, the pump was pre-run for 5 min to remove air bubbles, after which all vent valves were closed. The PBS mobile phase was then maintained at a constant rate of 1 mL/minute until the baseline stabilized. Subsequently, the sample was injected into the sampling valve, and its characteristics were monitored in real-time using the Waters Empower3 software. The operating parameters were as follows: flow rate of 1 mL/min, pressure of 3.3 MPa, temperature of 25 °C, and detection at a wavelength of 280 nm using UV detection.

### 2.7. ELISA

The 96-well NUNC microplates were coated with Trop2-ECD, EpCAM (Sino Biological, 10694-H08H) and another related antigen overnight at 4 °C. The solution in the well was then discarded and 2% milk was added to block for 2 h at room temperature. In the first well, the concentration of nanobody was 1000 nM, while the concentration of hRS7 was 250 nM. All antibodies were diluted serially three times. These solutions of the tested antibodies were added and allowed to react for 45 min at room temperature. Subsequently, HRP-conjugated secondary antibodies (GenScript, A01861, Piscataway Township, NJ, USA) were incubated for 30 min. The wells were washed at least 8 times with PT buffer, and TMB substrate was added for color development. To terminate the chromogen, 1.0 M H_3_PO_4_ (100 μL/well) was used. Finally, the OD_450_ was measured using the BioTek (Winooski, VT, USA) plate reader, and the EC_50_ value was calculated. The ELISA data were analyzed using GraphPad Prism 8.

### 2.8. Biolayer Interferometry (BLI)

BLI experiments were conducted using an Octet RED96 system (ForteBio, Fremont, CA, USA) and Protein A biosensors (ForteBio). The Trop2-ECD-Fc was immobilized by the specific binding of the chip to the Fc fragment. Ten anti-Trop2 nanobodies in a running buffer were used for the process of association and dissociation. The experiments were conducted in 96-well plates, each with a working volume of 200 μL, at a temperature of 30 °C and shaking at 1000 rpm. The reaction data was standardized using the Octet data analysis software (ForteBio).

### 2.9. Cell-Surface Binding by Flow Cytometry

Trop2-positive cells (NCI-N87 and MDA-MB-231) were resuspended in a FACS buffer (PBS containing 2% FBS) and were then plated into 96-well U-shaped bottom microplates, with 5 × 10^4^ cells per well. The cells were incubated for 1 h at 4 °C with VHH-Fc at a concentration of 500 nM. Following wash with FACS buffer, PE-labeled anti-human Fc secondary antibody (Abcam, 98596, Cambridge, UK) was added at 1000-fold dilution and incubated at 4 °C. After 30 min, the cells were washed and resuspended in FACS buffer. The mean fluorescent intensity (MFI) was detected using a Beckman Coulter flow cytometer. The binding data were then analyzed using FlowJo 10.4 and RStudio 2022.12.0 software.

### 2.10. Receptor Internalization Assay by Flow Cytometry

NCI-N87 and MDA-MB-231 cells were incubated with VHH-Fc on ice for 1 h, and subsequently, unbound antibody was removed by washing with FACS buffer. The cells were divided into two groups: one half of the cells were maintained on ice, while the other half were incubated at 37 °C for varying durations of 0.5, 1, 2, 4, and 6 h. Afterward, the cells were fixed in 4% paraformaldehyde for 20 min and subsequently stained with a PE-labeled anti-human Fc secondary antibody (Abcam, 98596). The Trop2 internalization rate was calculated as a percentage MFI loss at 37 °C relative to that on ice. The ability of nanobodies to interfere with the Trop2 cycling process was evaluated by changes in MFI over time. The data and *p*-values were analyzed by GraphPad Prism 8.

### 2.11. Cell Viability Assay

NCI-N87 and MDA-MB-231 cells were evenly distributed into 96-well cell culture plates at a density of 6000 cells per well and incubated overnight at 37 °C with 5% CO_2_. Serially diluted antibodies (1:3) were added to treat the cells for 5 days. After 4 days of incubation, the solution in the wells was discarded, and CCK8 solution was added to determine cell viability. The cells were then incubated in a 37 °C incubator for 2 h. The OD_450_ value was measured using BioTek plate reader to calculate the inhibition rate of antibodies on NCI-N87 and MDA-MB-231 cells. The calculation formula is inhibition rate (%) = [(Ac−As)/(Ac−Ab)] × 100%. Wherein As represents the experimental well (containing cells, medium, CCK-8, and nanobody); Ac: control well (containing cells, medium, and CCK-8); Ab: blank well (containing only medium and CCK-8). The data and *p*-values were analyzed using GraphPad Prism 8.

### 2.12. RNA Interference Analysis

NCI-N87 and MDA-MB-231 cells were incubated in a 6-well plate for 24 h until they reached 90% confluence. The Trop2-siRNA and transfection reagent (Lipofectamine LTX, ThermoFisher, Shanghai, China) were diluted following the kit instructions. Subsequently, the diluted siRNA was mixed with Lipofectamine LTX and incubated at room temperature for 30 min to form the siRNA-Lipofectamine LTX transfection complex. The mixture was then slowly dripped into the cells and further cultured at 37 °C with 5% CO_2_ for 48 h. Finally, the interference effect of Trop2-siRNA was validated through immunoblotting and was utilized in subsequent experiments.

### 2.13. Cell Migration Assay

Inoculate 200 uL of NCI-N87 cells with a density of 3 × 10^5^ cell/mL (serum-free 1640 medium) into the 8-μm pore-sized upper chamber of Transwell inserts (Corning Inc., Corning, NY, USA). Afterwards, N14 and N152 are added at a final concentration of 1.8 mM. As a chemoattractant, add 1640 medium with 10% FBS into the lower chamber of the Transwell. After incubation at 37 °C with 5% CO_2_ for 24 h, take the Transwell chambers and wash off the medium with PBS. Then, fix the migrated cells with 4% paraformaldehyde for 15 min, and subsequently stain with 0.5% crystal violet for 20 min. The upper non-migrated cells were gently wiped off with a cotton swab, and the remaining cells were observed and recorded under an inverted fluorescence microscope. Simultaneously, capture images of stained cells from randomly selected fields of view, facilitating cell migration analysis.

### 2.14. Epitope Analysis

The DNA sequences encoding chimeric Trop2-ECD proteins were generated by replacing the CRD (H27-L69), TY (T70-C145), CPD (D146-T274), and RCPD (Q237-Q252) of Trop2-ECD with the corresponding regions from the murine homolog. These DNA sequences were subsequently cloned into the pSCSTa vector, which contained a C-terminal Fc tag, and expressed by 293T cells. The chimeric proteins were expressed and purified using the identical methods as the VHH-Fc. Finally, four chimeric proteins, namely, hTrop2-mCRD, hTrop2-mTY, hTrop2-mCPD, and hTrop2-mRCPD, were obtained. Consistent with the ELISA assay method described above, hTrop2, mTrop2, and four chimeric proteins were coated on 96-well microplates. After antibody reaction, the HRP-conjugated mouse anti-human IgG Fab antibody (GenScript, A01855) and the HRP-conjugated secondary antibodies (GenScript, A01861) were added to label hRS7 and anti-Trop2 nanobodies, respectively. The color was developed using TMB, and finally the OD_450_ was measured by BioTek plate reader.

### 2.15. Co-Immunoprecipitation (Co-IP)

Protein G agarose beads (Sigma Aldrich, St. Louis, MO, USA) were used for preclearing the total cell lysates for 30 min at 4 °C. This was later treated with anti-Flag antibody post-preclearing, which was then incubated overnight at the same temperature on a tube rotator. The next day, we further added 20 μL of protein G beads and extended the incubation for 3 additional hours at 4 °C with end-to-end mixing. Centrifugation was subsequently used to pellet the beads which were washed three times using immunoprecipitation (IP) wash buffer (50 mM Tris/HCl, 2 mM EDTA, 100 mM NaCl, 0.5% NP40, and protease inhibitors). Lastly, the boiled immunoprecipitated protein complexes in Laemmli buffer were separated through SDS-PAGE for immunoblotting.

### 2.16. AlphaFold 3 Antibody–Antigen Modeling

Structural predictions for the N14 complex with either the Trop2-ECD monomer or the Trop2 *cis*-dimer were conducted using the AlphaFold 3 Server. The Trop2-ECD sequence was sourced from the PDB database, and the N14 sequence was obtained via sequencing. The CIF format results produced by AlphaFold 3 Server were subsequently imported into PyMOL for visualization analysis. The InterfaceResidues function in PyMOL identified residues at the interaction interface that are capable of forming hydrogen and ionic bonds within four angstroms.

### 2.17. Statistical Analysis

In this study, all data represent the final results as mean ± SEM. The GraphPad Prism software was utilized for statistical analysis. Student’s *t*-test was applied to analyze differences between the two samples. *p* < 0.05 was considered as significant and asterisks indicate the significant difference (ns, no significant; * *p* < 0.05; ** *p* < 0.01; *** *p* < 0.001).

## 3. Result

### 3.1. Screening and Characterization of Anti-Trop2 Nanobodies

**Figure 1:**
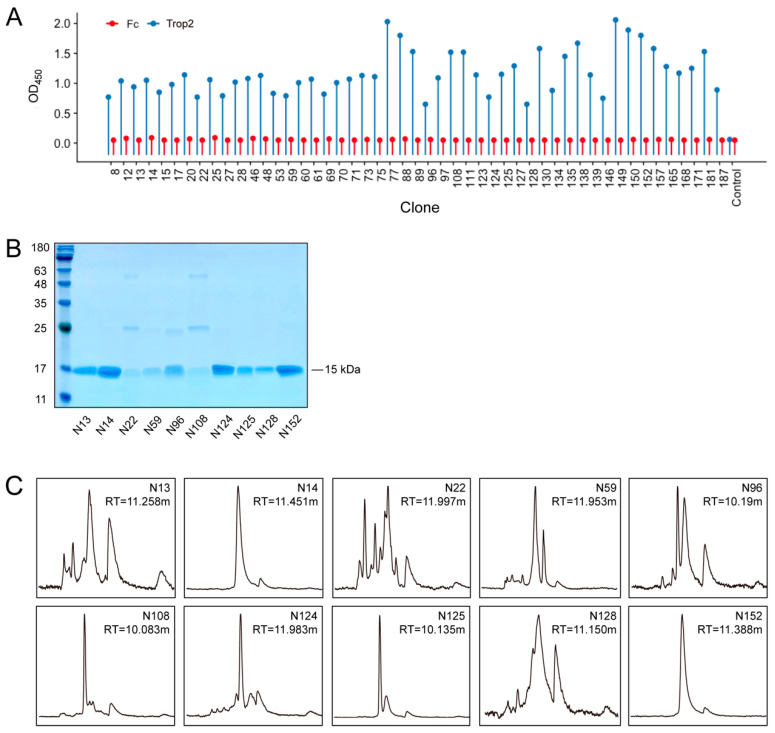
Screening and characterization of anti-Trop2 nanobodies. (**A**) Nanobodies were identified using phage ELISA. Fc fragment was employed as a negative control. (**B**) SDS-PAGE analysis of anti-Trop2 nanobodies. N13, N14, N124, N125, N128 and N152 exhibited good purity (approximately 95%). (**C**) SEC analysis of anti-Trop2 nanobodies. N14 and N152 are presented as single peaks and showed similar retention time (RT), demonstrating their great homogeneity.

To gain nanobodies against Trop2, we employed phage display technology and performed panning from a well-established synthetic nanobody library (ASyNAL, London, UK), with Trop2-ECD serving as the antigen. The quality control and diversity of ASyNAL library has been verified in our previous work [47]. We carried out sequencing of the positive clones identified in the phage ELISA and found that they originated from 10 unique sequences (N13, N14, N22, N59, N96, N108, N124, N125, N128, and N152), which were subsequently selected for expression (Figure 1A). The nanobodies were then purified through Ni-NTA columns, with their purity and homogeneity validated via SDS-PAGE and SEC analysis. The purity of six nanobodies (N13, N14, N124, N125, N128, and N152) was about 95%, while other nanobodies displayed impurities (Figure 1B). Unlike the results from SDS-PAGE, only N14 and N152 exhibited good homogeneity, as illustrated by the single peak in the SEC spectrum (Figure 1C). In contrast, the remaining nanobodies showed pronounced aggregation or fragments. In general, N14 and N152 possessed favorable properties among these 10 anti-Trop2 nanobodies.

### 3.2. N14 and N152 Exert Binding Specificity and Activity towards Trop2

**Figure 2:**
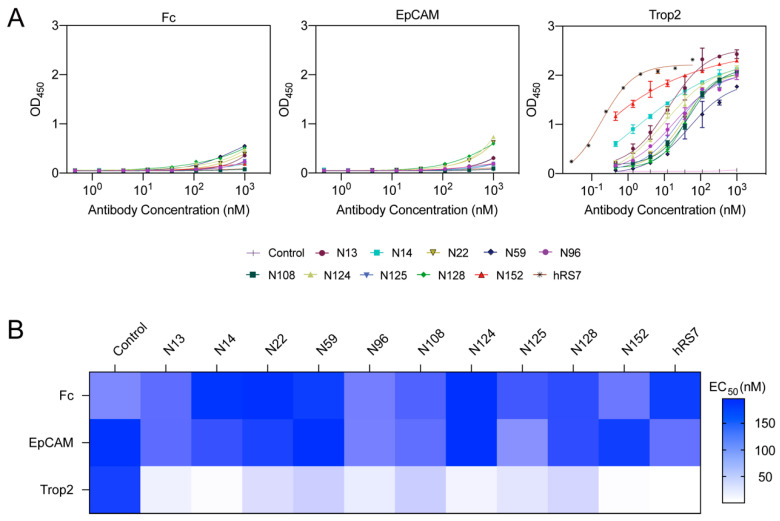
Specificity and binding activity of anti-Trop2 nanobodies. (**A**) The binding specificity of nanobodies identified by ELISA. hRS7 was used as a positive control, and an irrelevant nanobody (C5G2) served as a negative control. No antibodies showed an apparent reaction to Fc fragment and EpCAM. Both hRS7 and 10 anti-Trop2 nanobodies exhibited potent binding activity towards Trop2. The OD_450_ values were shown as the mean ± SEM (n = 3). (**B**) EC_50_ values of binding activity of nanobodies to Trop2. The values displayed in the heatmaps are the EC_50_ values of antibody binding to Fc fragment, EpCAM, and Trop2. The EC_50_ value of binding activity of N14 to Trop2 was 1.5 nM, while that of N152 was less than 1 nM.

Trop2 shares 49% homology and 67% similarity within amino acid sequence with EpCAM, the only known molecule from the same gene family of Trop2 [48]. Under normal conditions, EpCAM is extensively expressed on epithelial cells and is primarily involved in cell adhesion regulation [49]. Therefore, the ideal anti-Trop2 nanobodies should exclude the potential cross-reactivity with EpCAM and tag. Here, all anti-Trop2 nanobodies presented no response to EpCAM and Fc fragments but a pronounced association with Trop2, demonstrating their good binding specificity (Figure 2A). N14 and N152 exhibited the highest binding activity to Trop2, followed by N13, N96, and N124, while others displayed lower reactivity (Figure 2A). The EC_50_ value of N14 and N152 reached nearly nanomolar levels, which is similar to hRS7 (Figure 2B).

To further determine the affinity of anti-Trop2 nanobodies, we quantified their binding kinetics to recombinant Trop2 using BLI. The biosensor was loaded with the Fc-tagged Trop2 (Trop2-ECD-Fc) to capture the nanobodies. The results indicated that N14 and N152 also exhibited good binding strength to Trop2 (Table 1). In line with the aforementioned ELISA results, N14 demonstrated outstanding affinity (KD value, 18.0 nM) and low dissociation (Kdis value, 2.03 × 10^−4^ 1/s). However, N152 exhibited relatively lower affinity in BLI (KD value, 115.6 nM) potentially due to high dissociation properties (Kdis value, 1.95 × 10^−3^ 1/s). Taken together, based on the promising results of SDS-PAGE, SEC, ELISA, and BLI assays, N14 and N152 were selected for further functional investigation.

### 3.3. N14 and N152 Induce Receptor Internalization and Inhibit Receptor Recycling in Trop2-Positive Tumor Cells

**Figure 3:**
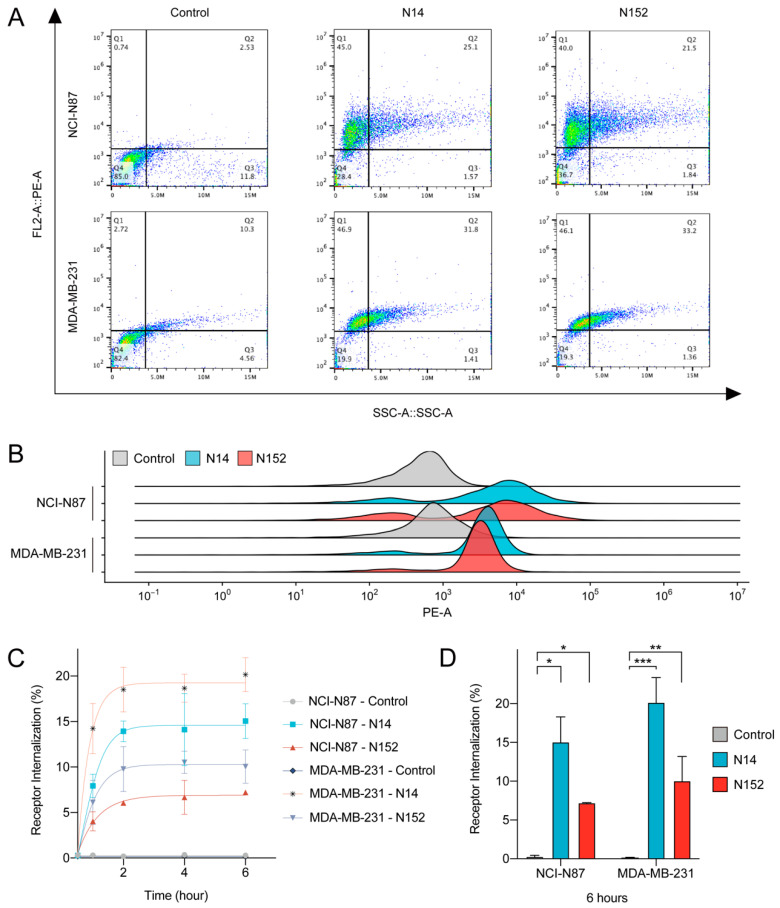
N14 and N152 bind to Trop2-positive tumor cells, mediate receptor internalization, and inhibit receptor recycling. (**A**,**B**) The cell-surface binding activity of N14 and N152 was quantified by flow cytometry in NCI-N87 and MDA-MB-231 cells. The majority of cells in the control group treated with the irrelevant nanobodies (C5G2) were unstained and migrated to the bottom left quadrant. In the experimental groups incubated with N14 and N152, the positive cells were located in the upper-left quadrant. Specifically, they represented 45% and 40% in NCI-N87 cells and 46.9% and 46.1% in MDA-MB-231 cells, respectively. The ridge plot characterizing mean fluorescence intensity is clearly shifted. (**C**,**D**) N14 and N152 mediate receptor internalization and inhibit receptor recycling in NCI-N87 cells. The irrelevant nanobody (C5G2) served as a negative control. N14 and N152 interfere Trop2 recycling on NCI-N87 and MDA-MB-231, resulting in sustained receptor internalization (2 h to 6 h). After 6 h, N14 and N152 mediate more significant receptor internalization in MDA-MB-231 cells, with internalization rates of 20.1% and 10.0% respectively, while in NCI-N87 they are 15% and 7.2%. The receptor internalization rate was shown as the mean ± SEM (n = 3). The statistical significance of the experiments was assessed using Student’s *t*-test (ns, no significant; * *p* < 0.05; ** *p* < 0.01; *** *p* < 0.001).

To evaluate the cell-surface binding of nanobodies towards Trop2-positive tumor cells, we fused nanobodies with human IgG Fc to construct VHH-Fc fusions and measured fluorescent signals in NCI-N87 and MDA-MB-231 cells by flow cytometry. Compared to control (C5G2), the tumor cells exhibited a significant signal shift after treatment with N14 and N152, providing evidence for the binding of N14 and N152 to Trop2 on the cell surface (Figure 3A,B). In contrast, when the same method was applied to Trop2-low/negative cells (SW620), no signal shift was observed, further confirming the specificity of N14 and N152 for Trop2-positive cells (Figure S1). Furthermore, the shift was more significant in NCI-N87 cells than in MDA-MB-231 cells, suggesting a higher levels of Trop2 in NCI-N87 cells. It has been well known that the approved Trop2-targeted ADC, SG, depends on the efficient internalization of its antibody backbone (hRS7) to deliver toxins into tumor cells and destroy them [5,50,51,52]. Likewise, some therapeutic antibodies could suppress tumor cells by rapid receptor internalization, inhibition of recycling, and lysosomal degradation. Here, we utilized flow cytometry to determine whether N14 and N152 could influence Trop2 internalization and recycling. NCI-N87 and MDA-MB-231 cells were treated with nanobodies for different periods. As shown in Figure 3C, N14 and N152 both elicited a much faster and higher levels of receptor internalization than the control. N14 treatment resulted in significantly greater internalization (approximately twofold) than N152 (Figure 3D). The Trop2 internalization rate was calculated based on the loss of MFI, therefore a high internalization rate indicates low levels of Trop2 on the cell surface. Continuous internalization activity (2 h to 6 h) induced by N14 and N152 means that they could disrupt receptor recycling, resulting in reduced levels of Trop2 on the cell surface. These results showed that N14 and N152 mediate receptor internalization and inhibit receptor recycling.

### 3.4. N14 and N152 Inhibit Cell Proliferation and Migration

**Figure 4:**
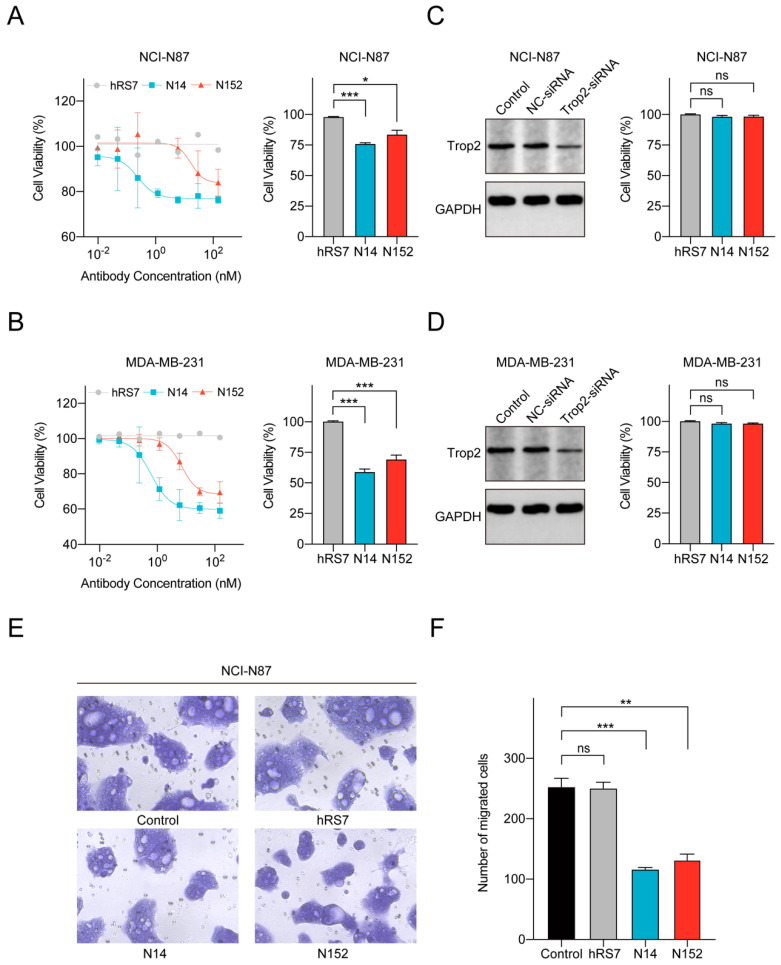
N14 and N152 directly inhibit cell proliferation and migration. (**A**,**B**) N14 and N152 inhibit the proliferation of NCI-N87 and MDA-MB-231 cells. The data is presented through proliferation inhibition curves (Left) and the bar graphs (Right) at the maximum concentration. Both N14 and N152 inhibit the proliferation of NCI-N87 (**A**) and MDA-MB-231 (**B**), compared to the control (hRS7). The growth inhibition effect mediated by N14 is consistently higher than that of N152. The cell viability was reported as the mean ± SEM (n = 3). (**C**,**D**) Left: Western blot analysis of Trop2 expression on NCI-N87 and MDA-MB-231 after RNA interference. The Trop2 expression on NCI-N87 (**C**) and MDA-MB-231 (**D**) was significantly reduced after the action of Trop2-siRNA, compared with the control and NC-siRNA. Right: The proliferation inhibitory effects of N14 and N152 on Trop2-depleted NCI-N87 and Trop2-depleted MDA-MB-231. After Trop2 knockdown, N14 and N152 lost their growth inhibitory effect on NCI-N87 (**C**) and MDA-MB-231 (**D**). The cell viability was reported as the mean ± SEM (n = 3). (**E**,**F**) Inhibition of cell migration by N14 and N152 in Transwell assay. The number of migrating cells decreased after treatment with N14 and N152, compared with the control (hRS7). N14 and N152 had a significant migration inhibitory effect on NCI-N87. The number of migrating cells was reported as the mean ± SEM (n = 3). All statistical significance of the experiments was assessed using Student’s *t*-test (ns, no significant; * *p* < 0.05; ** *p* < 0.01; *** *p* < 0.001).

Recent studies have reported that anti-Trop2 nanobodies were capable of inhibiting the migration of tumor cells but exhibited no efficacy in inhibiting tumor cell growth [46]. We next assessed whether the Trop2 internalization would translate into antiproliferation efficacy in Trop2-positive tumor cells. NCI-N87 and MDA-MB-231 cells were treated with N14 and N152 for 5 days and measured the cell viability with a Cell Counting Kit-8 (CCK-8). Consistent with the published data, the control (hRS7) had no inhibitory effects on cell proliferation. In contrast, N14 and N152 showed a pattern of concentration-dependent inhibition of cell viability (Figure 4A,B). In both NCI-N87 and MDA-MB-231, N14 demonstrated greater growth inhibition than N152 (Figure 4A,B). Notably, a more significant anti-tumor activity was observed in MDA-MB-231, which may be associated with a greater degree of receptor internalization. Moreover, the knockdown of Trop2 in NCI-N87 cells via siRNA rendered both N14 and N152 ineffective in inhibiting cell proliferation (Figure 4C). Similar results were obtained in Trop2-depleted MDA-MB-231 cells (Figure 4D). Correspondingly, N14 and N152 do not affect the proliferation of Trop2-low/negative tumor cells (SW620) (Figure S2). These findings suggest that the anti-tumor activity of these nanobodies depends on the presence of Trop2 in tumor cells. We then assessed the effects of N14 and N152 on the migration of Trop2-positive tumor cells through Transwell assay. After treatment with N14 and N152, there was a notable decrease in the number of NCI-N87 cells migrating to the lower chamber, indicating that both N14 and N152 significantly impeded the migration of Trop2-positive cancer cells, with N14 showing superior inhibitory activity (Figure 4E,F). Taken together, these results revealed that N14 and N152 can effectively inhibit the growth and migration of Trop2-positive cancer cells.

### 3.5. N14 and N152 Recognize Multiple Domains of Trop2-ECD and Inhibit Trop2 Dimerization

**Figure 5:**
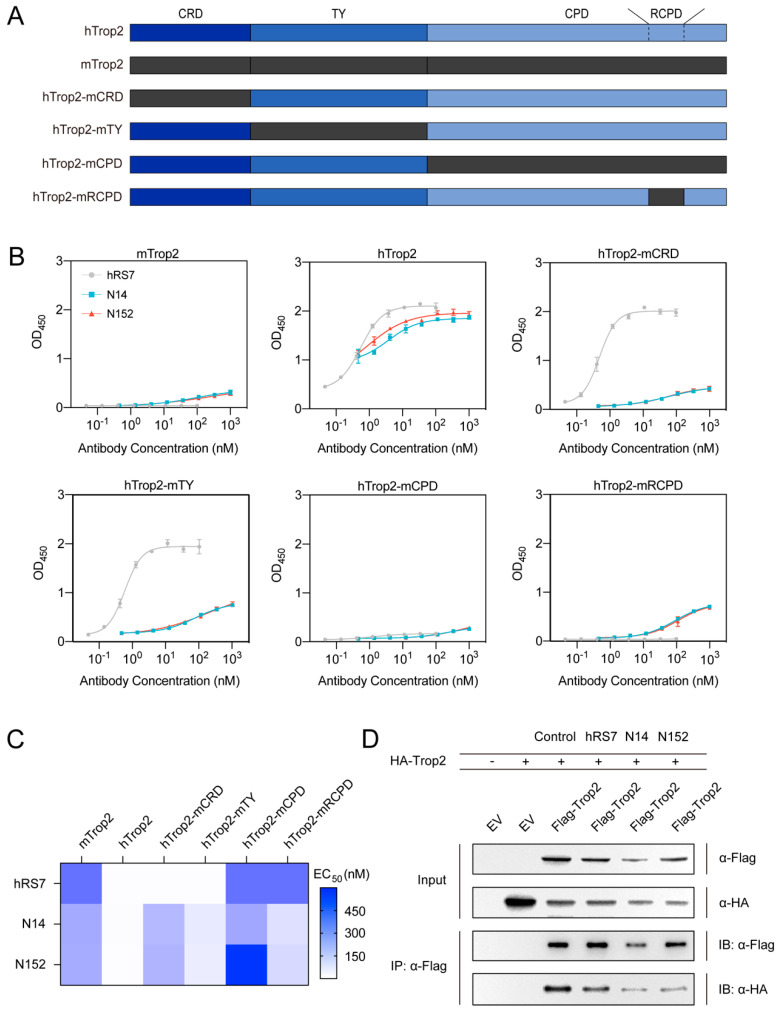
N14 and N152 bind multiple domains of Trop2-ECD and inhibit Trop2 dimerization. (**A**) Schematic diagram of domain- or loop-substituted Trop2-ECD constructs. hTrop2-mCRD, hTrop2-mTY, hTrop2-mCPD, and hTrop2-mRCPD corresponded to the CRD (H27-L69), TY (T70-C145), CPD (D146-T274), and RCPD (Q237-Q252) of Trop2 replaced by that of murine homolog, respectively. All chimeric and wild-type Trop2 proteins were expressed in the 293T system. (**B**) ELISA analysis of the key domains of interaction between Trop2 and nanobodies. hRS7 recognized the RCPD, an exposed loop within the CPD of Trop2-ECD. N14 and N152 recognize multiple domains of Trop2-ECD. The OD_450_ values are reported as the mean ± SEM (n = 3). (**C**) Heatmap of the epitope-mapping results observed for the anti-Trop2 nanobodies. The EC_50_ value of nanobodies against different chimeric Trop2-ECD proteins was measured and displayed as a heatmap. (**D**) N14 and N152 inhibit Trop2 dimerization. Cells solely transfected with EV (lane 1) or co-transfected with EV (empty vector) and HA-Trop2 (lane 2) served as negative controls for dimerization. The cells co-transfected with Flag-Trop2 and HA-Trop2 as the experimental group (lanes 3-6). The cells treated with hRS7, N14, and N152 were lysed four hours later (Lanes 4, 5, and 6, respectively), with cells receiving no treatment serving as negative controls (Lane 3). The total lysate (used as an input) was probed with anti-Flag antibody and anti-HA antibody (Panels 1 and 2, respectively). The presence of Flag-Trop2 Please check that intended meaning is retained.in respective IP lanes was confirmed by probing with anti-Flag antibody (panel 3) and immune complexes pulled down by anti-Flag antibody were probed with anti-HA antibody for detection of co-immunoprecipitated HA-tagged monomers (panel 4).

Trop2-ECD consists of three subdomains (CRD, TY, and CPD), which play critical roles in oligomeric complexity [34,53]. To determine the binding pattern of N14 and N152, we replaced subdomains of human Trop2-ECD (hTrop2-ECD) with those of mouse homolog to generate chimeric Trop2-ECD proteins (hTrop2-mCRD, hTrop2-mTY, and hTrop2-mCPD) (Figure 5A). In addition, an exposed loop (Q237-Q252) within CPD was substituted to generate the hTrop2-mRCPD, which has been identified as binding regions for hRS7 (Figure 5A) [34]. Subsequently, we determined the reactivity of anti-Trop2 nanobodies with each chimeric protein using ELISA. The results showed that the control (hRS7), N14, and N152 had no response to mTrop2. hTrop2-mCPD and hTrop2-mRCPD reduced the binding activity of hRS7, demonstrating its binding epitopes were located in RCPD, which is identical to the previous research (Figure 5B) [34]. Notably, the reactivity of N14 and N152 towards the four chimeric proteins was considerably diminished compared to their binding activity to hTrop2. Especially, N14 and N152 almost lost association with hTrop2-mCRD and hTrop2-mCPD, while retaining modest binding activity to hTrop2-mTY and hTrop2-mRCPD (Figure 5B,C). These results suggested that the N14 and N152 can bind to all three subdomains of Trop2-ECD, in which CPD and CRD make relatively important contributions. Previous studies have revealed that the TY and CPD domains of Trop2-ECD are involved in the assembly of Trop2 dimers [34]. Given the unique binding pattern of N14 and N152, which simultaneously interact with all three subdomains of Trop2-ECD, these nanobodies could inhibit Trop2 dimerization. We next performed co-IP experiments to investigate whether N14 and N152 can inhibit the assembly of Trop2 dimers. We engineered two Trop2 constructs (Flag-Trop2 and HA-Trop2) and co-expressed them in HEK293 cells. As expected, treatment with hRS7 did not alter the levels of HA-Trop2 relative to Flag-Trop2 in the cell lysate, indicating that hRS7 does not inhibit Trop2 dimerization. Remarkably, following treatment with N14 and N152, the HA-Trop2 levels significantly decreased, demonstrating their potent inhibitory effect on Trop2 dimerization (Figure 5D). These unique binding patterns and the ability to inhibit Trop2 dimerization are distinctive features of N14 and N152, not observed in previously reported Trop2-targeted antibodies.

### 3.6. N14 Binds to the Site on Cis-Interface

**Figure 6:**
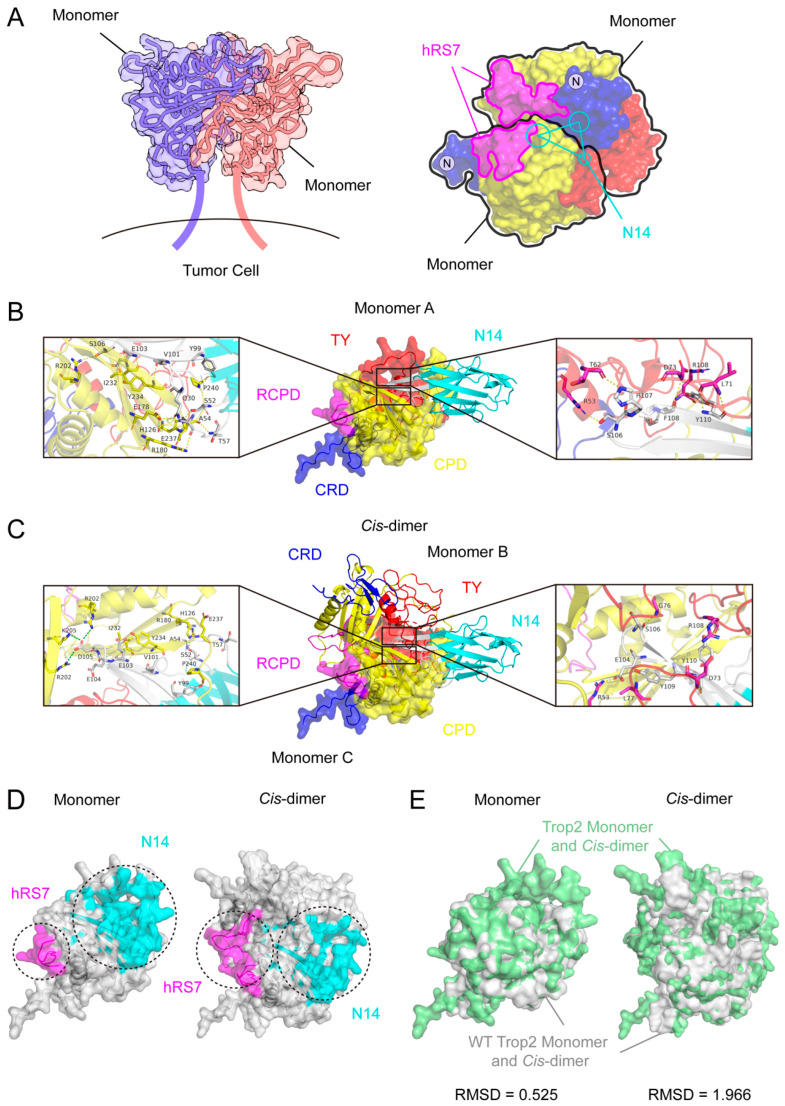
The binding sites of N14. (**A**) Left: Cartoon diagram illustrating the Trop2 *cis-*dimers. Right: The potential binding sites for N14 at *cis*-interfaces. The structural model of the *cis*-dimer with PDB ID 7E5N. The potential binding epitopes of N14 are indicated by the cyan lines, while the magenta circle encloses the binding epitopes (RCPD) of hRS7. The black lines represent two Trop2 monomers in *cis*-dimers. (**B**) The interaction between N14 and the Trop2-ECD monomer. The white segments in N14 represent the three CDRs. The enlarged area displays the details of the interaction interface. Residues that participate in hydrogen bond interactions are shown as sticks and labeled. Hydrogen bonds are shown as dashed yellow lines. (**C**) The interactions between N14 and the Trop2 *cis-*dimer. One subunit (Monomer B) in the *cis*-dimer structure is the cartoon representation, and the other subunit (Monomer C) presents a translucent surface format. The white segments in N14 represent the three CDRs. The enlarged area displays the details of the interaction interface. Residues that participate in hydrogen bond interactions are shown as sticks and labeled. Hydrogen bonds are shown as dashed yellow lines. Salt bonds are shown as dashed green lines. (**D**) The binding regions of hRS7 and N14 on the Trop2-ECD monomer (left) and *cis*-dimer (right). The control antibodies hRS7 are indicated in magenta, while the N14 are depicted in cyan. (**E**) Left: Compares the WT Trop2-ECD monomer (in gray) with the N14-bound Trop2-ECD monomer (in green). The RMSD was 0.525 Å. Right: Contrasts the WT *cis*-dimer (in gray) with the N14-bound *cis*-dimer (in green). The RMSD was 1.966 Å.

Epitope analysis results indicate that N14 and N152 bind to all three subdomains of the Trop2-ECD. This unique binding pattern inhibits Trop2 dimerization. Thus, it was reasonable to infer that N14 and N152 bind to the conformational epitopes at the interface of the Trop2 dimer. Due to the limited length of the CDRs in nanobodies, it is theoretically impossible for N14 and N152 to span all three subdomains of the Trop2-ECD monomer and *trans*-dimer. Interestingly, when it comes to *cis-*dimers, the subdomains of two Trop2-ECD molecules come closer, making the unique binding mode of N14 and N152 feasible (Figure 6A).

Recently, the introduction of AlphaFold 3 has sparked a revolution in modeling the structures of proteins and their interactions. The AlphaFold 3 model has significantly enhanced the precision of joint structure prediction for complexes, including proteins, nucleic acids, small molecules, ions, and modified residues [54] given the similar binding modes of N14 and N152 and considering that N14 surpasses N152 in mediating receptor internalization as well as inhibiting the proliferation and migration of Trop2-positive tumor cells. We have further utilized AlphaFold 3 to predict the complex structures of N14 with both Trop2-ECD monomers and *cis*-dimers. Additionally, we employed PyMOL to analyze the critical residues involved in their interactions.

The results show that, in the Trop2-ECD monomer structure (Monomer A), the CDRs of N14 mediates recognition through thirteen interactions with CPD and TY, comprising eight hydrogen bonds with CPD (H126^A^-S52^N14^, E178^A^-Q30^N14^, R180^A^-A54^N14^, R202^A^-S106^N14^, I232^A^-E103^N14^, Y234^A^-V101^N14^, E237^A^-T57^N14^, and P240^A^-Y99^N14^) and five hydrogen bonds with TY (R53^A^-S106^N14^, T62^A^-H107^N14^, L71^A^-Y110^N14^, D73^A^-F108^N14^, and R108^A^-Y110^N14^) (Figure 6B).

In the *cis*-dimer structure, the CDRs of N14 engages with two distinct Trop2-ECD monomers (Monomer B and Monomer C), with the binding sites situated at the *cis*-interface (Figure 6C,D). Specifically, in Monomer B, the CDRs of N14 binds to CPD, resulting in seven hydrogen bonds (H126^B^-S52^N14^, R180^B^-A54, I232^B^-E103^N14^, Y234^B^-E103^N14^, Y234^B^-V101^N14^, E237^B^-T57^N14^, and P240^B^-Y99^N14^) and a salt bridge (R202^B^-D105^N14^) and also engages with TY, forming two hydrogen bonds (G76^B^-S106^N14^ and R108^B^-Y110^N14^). In Monomer C, similar interactions occur with CPD, forming two salt bridges (R202^C^-D105^N14^ and K205^C^-D105^N14^), and with TY, forming two hydrogen bonds (D73^C^-Y110^N14^ and L77^C^-Y109^N14^) and a salt bridge (R53^C^-E104^N14^) (Figure 6C).

Subsequently, the structures of the wild-type (WT) Trop2-ECD monomer and *cis*-dimer (PDB ID: 7E5N) were aligned with their N14-bound counterparts. The alignment revealed that N14 binding does not significantly alter the conformation of the Trop2-ECD monomer, with an RMSD of 0.525 Å (Figure 6E). Interestingly, the N14 interaction resulted in conformational changes in the *cis-*dimer, as indicated by an RMSD of 1.966 Å (Figure 6E). This conformational alteration may contribute to the inhibition of Trop2 dimerization by these anti-Trop2 nanobodies.

## 4. Discussion

Over the past five years, Trop2 has been established as a highly attractive target in multiple tumor types, especially TNBC. However, side effects and drug resistance remain a major challenge for current Trop2-targeted therapies. Recent structural studies revealed that Trop2-ECD can assemble into *cis-* or *trans-*dimers, which potentially serve as key switches for activating oncogenic signaling [34,55]. hRS7, the antibody backbone of SG, lacks a direct tumor inhibitory effect, partly due to its binding sites far from the dimerization interface of Trop2 [34,35]. Similar to hRS7, the other existing anti-Trop2 antibodies mainly bind to immunodominant epitopes at Trop2-CPD. This potentially explains their limited ability to prevent Trop2 dimerization or inhibit proliferation effectively. Therefore, we hypothesized that targeting the novel epitopes within the dimerization interface might block the ordered assembly of Trop2, thereby providing an alternative strategy to inhibit the oncogenic functions of Trop2.

Given the unique advantages of nanobodies to recognize cryptic epitopes, we sought to screen nanobodies that bind to sites beyond the immunodominant epitopes and exhibit therapeutic potential from the synthetic nanobody library. Two Trop2-specific nanobodies, N14 and N152, were obtained after multiple rounds of panning. They both mediate receptor internalization and exhibit higher antitumor efficacy than hRS7. Epitope analysis revealed that N14 and N152 demonstrate a unique binding pattern, associating with all three subdomains of Trop2-ECD. Further analysis using Alphafold 3 and PyMOL of the potential binding sites for N14 suggests that N14 may bind at the interface of *cis*-dimerization. Additionally, the binding of N14 alter the conformation of the *cis*-dimer (Figure 6E). Although these results explain the inhibition of Trop2 dimer assembly by N14 (Figure 5D). However, N14 do not bind to all three subdomains of the Trop2-ECD in *cis*-dimers. This finding contradicts the results from the epitope identification experiments mentioned earlier (Figure 5B). Consequently, to accurately ascertain the binding sites of N14, further investigation employing techniques such as cryo-electron microscopy, structural biology, and predictive modeling is essential.

Although N14 and N152 share similar functions and binding modalities, their affinity was slightly different (Table 1). Compared to N152, N14 demonstrated a lower dissociation rate, resulting in a higher affinity. This feature allowed stable saturation activity for N14 to Trop2-positive tumor cells for a longer time. Combined with its ability to induce receptor internalization, N14 might be more suitable than N152 as a nanobody-based tool for drug delivery [56,57,58]. Currently, Trop2 has been employed in noninvasive imaging for tumor detection and treatment monitoring. A bispecific antibody that targets Trop2 and histamine-succinyl-glycine (HSG) enables specific imaging of tumor cells through binding with radioactively labeled synthetic peptides [59,60,61,62,63]. Based on the advantages of nanobodies in imaging, we proposed that N152 may serve as a tool for targeted imaging of Trop2-positive tumor patients. Due to the relatively rapid dissociation rate, N152 has the potential to maintain a balance between sufficient target retention and efficient clearance from non-target tissues, resulting in enhanced target-to-background ratios and improved imaging sensitivity and specificity [64,65].

Recent studies have reported that anti-Trop2 nanobodies could effectively suppress tumor cell migration but did not inhibit cell proliferation [46]. Our anti-Trop2 nanobodies not only mediated pronounced receptor internalization but also induced a significant decrease in cell viability and migration of Trop2-positive tumor cells. This functional discrepancy between different nanobodies is mainly due to inconsistencies in the binding epitopes of the nanobodies. Despite N14 and N152 demonstrating greater antitumor efficacy than previously reported anti-Trop2 nanobodies, their affinity was lower than that of hRS7, restricting the potential applications in Trop2-targeted therapeutics. Therefore, it is still necessary to engineer N14 and N152 for enhanced affinity and more potent antitumor activity.

## 5. Conclusions

We obtained two anti-Trop2 nanobodies, N14 and N152, from the ASyNAL library. They could bind to Trop2 with high affinity and specificity. Importantly, they are capable of effectively mediating receptor internalization, disrupting receptor recycling, and inhibiting cell growth and migration in Trop2-positive tumor cells. Epitope analysis has demonstrated that these anti-Trop2 nanobodies possess a unique binding pattern, interacting with all three subdomains of Trop2-ECD, with binding sites likely located at the interface of the Trop2 *cis*-dimer. It is noteworthy that the inhibition of Trop2 dimer assembly by N14 and N152 has not been observed in other reported Trop2-targeting antibodies. In summary, our findings indicate that the novel anti-Trop2 nanobodies hold considerable potential as targeted therapeutics for Trop2-positive tumors.

## Figures and Tables

**Table 1:** The affinities of anti-Trop2 nanobodies binding to Trop2-ECD.

	Kon (1/Ms)	Kdis (1/s)	KD (nM)
N13	(4.45 ± 0.05) × 10^3^	(2.34 ± 0.08) × 10^−4^	52.6 ± 1.79
N14	(1.14 ± 0.04) × 10^4^	(2.03 ± 0.09) × 10^−4^	18.0 ± 0.80
N22	(1.44 ± 0.02) × 10^4^	(2.12 ± 0.03) × 10^−3^	147.3 ± 3.02
N59	(1.29 ± 0.06) × 10^4^	(2.74 ± 0.10) × 10^−3^	213.0 ± 12.6
N96	(1.28 ± 0.03) × 10^4^	(1.62 ± 0.05) × 10^−3^	126.6 ± 5.25
N108	(4.27 ± 0.07) × 10^3^	(5.93 ± 0.12) × 10^−4^	138.7 ± 3.52
N124	(1.10 ± 0.03) × 10^4^	(1.28 ± 0.05) × 10^−3^	115.6 ± 6.07
N125	N/A	N/A	N/A
N128	(4.35 ± 0.13) × 10^3^	(4.21 ± 0.22) × 10^−4^	96.8 ± 5.70
N152	(1.69 ± 0.04) × 10^4^	(1.95 ± 0.09) × 10^−3^	115.6 ± 5.88

## Data Availability

Data will be made available on request.

## Supplementary Materials

The following supporting information can be downloaded at: https://www.mdpi.com/article/10.3390/pharmaceutics16101255/s1. Figure S1. Flow cytometry of N14 and N152 with Trop2-low/negative tumor cells (SW620). Figure S2. Cell viability assays of Trop2-low/negative tumor cells (SW620) treated with N14 and N152.

## Abbreviations

ADC: antibody drug conjugates; BLI, biolayer interferometry; CPD, cysteine-poor domain; CRD, cysteine-rich domain; E.coli, Escherichia coli; ECD, extracellular domain; ELISA, enzyme-linked immunosorbent assay; EpCAM, epithelial cell adhesion molecule; Fc, fragment crystallizable region; FDA, Food and Drug Administration; IgG, immunoglobulin G; MFI, mean fluorescence intensity; mTNBC, metastatic triple-negative breast cancer; ORR, objective response rate; PE, phycoerythrin; SDS-PAGE, sodium dodecyl sulfate polyacrylamide gel electrophoresis; SEC, Size-exclusion chromatography; SEM, standard error of the mean; SG, sacituzumab govitecan; Trop2, trophoblast cell-surface antigen 2; TNBC, triple-negative breast cancer; TY, thyroglobulin type-1; VHH, heavy chain variable region.

## References

[1] Tsukahara Y., Tanaka M., Miyajima A. (2011). TROP2 expressed in the trunk of the ureteric duct regulates branching morphogenesis during kidney development. PLoS ONE.

[2] Moretto R., Germani M.M., Giordano M., Conca V., Proietti A., Niccoli C., Pietrantonio F., Lonardi S., Tamburini E., Zaniboni A. (2023). Trop-2 and Nectin-4 immunohistochemical expression in metastatic colorectal cancer: Searching for the right population for drugs’ development. Br. J. Cancer.

[3] Chen C., Chao Y., Zhang C., Hu W., Huang Y., Lv Y., Liu B., Ji D., Liu M., Yang B. (2023). TROP2 translation mediated by dual m(6)A/m(7)G RNA modifications promotes bladder cancer development. Cancer Lett..

[4] Cubas R., Li M., Chen C., Yao Q. (2009). Trop2: A possible therapeutic target for late stage epithelial carcinomas. Biochim. Biophys. Acta (BBA)-Rev. Cancer.

[5] Liu X., Deng J., Yuan Y., Chen W., Sun W., Wang Y., Huang H., Liang B., Ming T., Wen J. (2022). Advances in Trop2-targeted therapy: Novel agents and opportunities beyond breast cancer. Pharmacol. Ther..

[6] Basu A., Goldenberg D.M., Stein R. (1995). The epithelial/carcinoma antigen EGP-1, recognized by monoclonal antibody RS7-3G11, is phosphorylated on serine 303. Int. J. Cancer.

[7] Berridge M.J. (1995). Calcium signalling and cell proliferation. BioEssays.

[8] Cubas R., Zhang S., Li M., Chen C., Yao Q. (2010). Trop2 expression contributes to tumor pathogenesis by activating the ERK MAPK pathway. Mol. Cancer.

[9] Lupu V.D., Kaznacheyeva E., Krishna U.M., Falck J.R., Bezprozvanny I. (1998). Functional coupling of phosphatidylinositol 4,5-bisphosphate to inositol 1,4,5-trisphosphate receptor. J. Biol. Chem..

[10] Stoyanova T., Goldstein A.S., Cai H., Drake J.M., Huang J., Witte O.N. (2012). Regulated proteolysis of Trop2 drives epithelial hyperplasia and stem cell self-renewal via β-catenin signaling. Genes Dev..

[11] Nakatsukasa M., Kawasaki S., Yamasaki K., Fukuoka H., Matsuda A., Tsujikawa M., Tanioka H., Nagata-Takaoka M., Hamuro J., Kinoshita S. (2010). Tumor-associated calcium signal transducer 2 is required for the proper subcellular localization of claudin 1 and 7: Implications in the pathogenesis of gelatinous drop-like corneal dystrophy. Am. J. Pathol..

[12] Hou J., Lv A., Deng Q., Zhang G., Hu X., Cui H. (2019). TROP2 promotes the proliferation and metastasis of glioblastoma cells by activating the JAK2/STAT3 signaling pathway. Oncol. Rep..

[13] Lin J.C., Wu Y.Y., Wu J.Y., Lin T.C., Wu C.T., Chang Y.L., Jou Y.S., Hong T.M., Yang P.C. (2012). TROP2 is epigenetically inactivated and modulates IGF-1R signalling in lung adenocarcinoma. EMBO Mol. Med..

[14] Tang G., Tang Q., Jia L., Chen Y., Lin L., Kuai X., Gong A., Feng Z. (2019). TROP2 increases growth and metastasis of human oral squamous cell carcinoma through activation of the PI3K/Akt signaling pathway. Int. J. Mol. Med..

[15] Guo X., Zhu X., Zhao L., Li X., Cheng D., Feng K. (2017). Tumor-associated calcium signal transducer 2 regulates neovascularization of non-small-cell lung cancer via activating ERK1/2 signaling pathway. Tumour Biol..

[16] Iwamoto S., Mori Y., Yamashita T., Ojima K., Akita K., Togano S., Kushiyama S., Yashiro M., Yatera Y., Yamaguchi T. (2023). Trophoblast cell surface antigen-2 phosphorylation triggered by binding of galectin-3 drives metastasis through down-regulation of E-cadherin. J. Biol. Chem..

[17] Jiang H., Qin X., Wang Q., Xu Q., Wang J., Wu Y., Chen W., Wang C., Zhang T., Xing D. (2021). Application of carbohydrates in approved small molecule drugs: A review. Eur. J. Med. Chem..

[18] Ma Q., Jiang H., Ma L., Zhao G., Xu Q., Guo D., He N., Liu H., Meng Z., Liu J. (2023). The moonlighting function of glycolytic enzyme enolase-1 promotes choline phospholipid metabolism and tumor cell proliferation. Proc. Natl. Acad. Sci. USA.

[19] Liu X., Ma L., Li J., Sun L., Yang Y., Liu T., Xing D., Yan S., Zhang M. (2024). Trop2-targeted therapies in solid tumors: Advances and future directions. Theranostics.

[20] Petrylak D.P., Tagawa S.T., Jain R.K., Bupathi M., Balar A.V., Rezazadeh A., George S., Palmbos P.L., Nordquist L.T., Davis N.B. (2023). Primary analysis of TROPHY-U-01 cohort 2, a phase 2 study of sacituzumab govitecan (SG) in platinum (PT)-ineligible patients (pts) with metastatic urothelial cancer (mUC) that progressed after prior checkpoint inhibitor (CPI) therapy. J. Clin. Oncol..

[21] Rugo H.S., Bardia A., Marmé F., Cortes J., Schmid P., Loirat D., Tredan O., Ciruelos E., Dalenc F., Pardo P.G. (2022). Primary results from TROPiCS-02: A randomized phase 3 study of sacituzumab govitecan (SG) versus treatment of physician’s choice (TPC) in patients (Pts) with hormone receptor–positive/HER2-negative (HR+/HER2-) advanced breast cancer. J. Clin. Oncol..

[22] Tagawa S.T., Balar A.V., Petrylak D.P., Kalebasty A.R., Loriot Y., Fléchon A., Jain R.K., Agarwal N., Bupathi M., Barthelemy P. (2021). TROPHY-U-01: A Phase II Open-Label Study of Sacituzumab Govitecan in Patients With Metastatic Urothelial Carcinoma Progressing After Platinum-Based Chemotherapy and Checkpoint Inhibitors. J. Clin. Oncol..

[23] Tagawa S.T., Faltas B.M., Lam E.T., Saylor P.J., Bardia A., Hajdenberg J., Morgans A.K., Lim E.A., Kalinsky K., Simpson P.S. (2019). Sacituzumab govitecan (IMMU-132) in patients with previously treated metastatic urothelial cancer (mUC): Results from a phase I/II study. J. Clin. Oncol..

[24] Garon E., Johnson M., Lisberg A., Spira A., Yamamoto N., Heist R., Sands J., Yoh K., Meric-Bernstam F., Kitazono S. (2021). MA03.02 TROPION-PanTumor01: Updated Results From the NSCLC Cohort of the Phase 1 Study of Datopotamab Deruxtecan in Solid Tumors. J. Thorac. Oncol..

[25] Bardia A., Messersmith W., Kio E., Berlin J., Vahdat L., Masters G., Moroose R., Santin A., Kalinsky K., Picozzi V. (2021). Sacituzumab govitecan, a Trop-2-directed antibody-drug conjugate, for patients with epithelial cancer: Final safety and efficacy results from the phase I/II IMMU-132-01 basket trial. Ann. Oncol..

[26] Kalinsky K., Diamond J., Vahdat L., Tolaney S., Juric D., O’SHaughnessy J., Moroose R., Mayer I., Abramson V., Goldenberg D. (2020). Sacituzumab govitecan in previously treated hormone receptor-positive/HER2-negative metastatic breast cancer: Final results from a phase I/II, single-arm, basket trial. Ann. Oncol..

[27] Bardia A., Mayer I.A., Vahdat L.T., Tolaney S.M., Isakoff S.J., Diamond J.R., O’Shaughnessy J., Moroose R.L., Santin A.D., Abramson V.G. (2019). Sacituzumab Govitecan-hziy in Refractory Metastatic Triple-Negative Breast Cancer. N. Engl. J. Med..

[28] Bardia A., Hurvitz S.A., Tolaney S.M., Loirat D., Punie K., Oliveira M., Brufsky A., Sardesai S.D., Kalinsky K., Zelnak A.B. (2021). Sacituzumab Govitecan in Metastatic Triple-Negative Breast Cancer. N. Engl. J. Med..

[29] Krop I., Juric D., Shimizu T., Tolcher A., Spira A., Mukohara T., Lisberg A.E., Kogawa T., Papadopoulos K.P., Hamilton E. (2022). Abstract GS1-05: Datopotamab deruxtecan in advanced/metastatic HER2- breast cancer: Results from the phase 1 TROPION-PanTumor01 study. Cancer Res..

[30] Shaffer C. (2021). Trop2 deal heats up antibody–drug conjugate space in cancer. Nat. Biotechnol..

[31] Coates J.T., Sun S., Leshchiner I., Thimmiah N., Martin E.E., McLoughlin D., Danysh B.P., Slowik K., Jacobs R.A., Rhrissorrakrai A. (2021). Parallel Genomic Alterations of Antigen and Payload Targets Mediate Polyclonal Acquired Clinical Resistance to Sacituzumab Govitecan in Triple-Negative Breast Cancer. Cancer Discov..

[32] Liu X., Li J., Deng J., Zhao J., Zhao G., Zhang T., Jiang H., Liang B., Xing D., Wang J. (2023). Targeting Trop2 in solid tumors: A look into structures and novel epitopes. Front. Immunol..

[33] Kamble P.R., Patkar S.R., Breed A.A., Pathak B.R. (2021). N-glycosylation status of Trop2 impacts its surface density, interaction with claudin-7 and exosomal release. Arch. Biochem. Biophys..

[34] Sun M., Zhang H., Jiang M., Chai Y., Qi J., Gao G.F., Tan S. (2021). Structural insights into the cis and trans assembly of human trophoblast cell surface antigen 2. iScience.

[35] Han C., Perrone E., Zeybek B., Bellone S., Tymon-Rosario J., Altwerger G., Menderes G., Feinberg J., Haines K., Karger M.E.M. (2020). In vitro and in vivo activity of sacituzumab govitecan, an antibody-drug conjugate targeting trophoblast cell-surface antigen 2 (Trop-2) in uterine serous carcinoma. Gynecol. Oncol..

[36] Truong A., Feng N., Sayegh D., Mak B., O’Reilly K., Fung S.W., Ceric N., Hahn S., Pereira D., Findlay H. (2007). AR47A6.4.2, a functional naked monoclonal antibody targeting Trop-2, demonstrates in vivo efficacy in human pancreatic, colon, breast and prostate cancer models. Mol. Cancer Ther..

[37] Trerotola M., Guerra E., Ali Z., Aloisi A.L., Ceci M., Simeone P., Acciarito A., Zanna P., Vacca G., D’AMore A. (2021). Trop-2 cleavage by ADAM10 is an activator switch for cancer growth and metastasis. Neoplasia.

[38] Alberti S., Miotti S., Stella M., Klein C.E., Fornaro M., Menard S., Colnaghi M.I. (1992). Biochemical characterization of Trop-2, a cell surface molecule expressed by human carcinomas: Formal proof that the monoclonal antibodies T16 and MOv-16 recognize Trop-2. Hybridoma.

[39] Ikeda M., Yamaguchi M., Kato K., Nakamura K., Shiina S., Ichikawa-Ando T., Misaka H., Myojo K., Nakamura K., Sugimoto Y. (2015). Pr1E11, a novel anti-TROP-2 antibody isolated by adenovirus-based antibody screening, recognizes a unique epitope. Biochem. Biophys. Res. Commun..

[40] Kaufmann R., Hainzl A., Sterry W., Alberti S., Klein C.E. (1994). In vivo targeting of integrin receptors in human skin xenografts by intravenously applied antibodies. Arch. Dermatol. Res..

[41] Stepan L.P., Trueblood E.S., Hale K., Babcook J., Borges L., Sutherland C.L. (2011). Expression of Trop2 cell surface glycoprotein in normal and tumor tissues: Potential implications as a cancer therapeutic target. J. Histochem. Cytochem..

[42] Trerotola M., Cantanelli P., Guerra E., Tripaldi R., Aloisi A.L., Bonasera V., Lattanzio R., de Lange R., Weidle U.H., Piantelli M. (2013). Upregulation of Trop-2 quantitatively stimulates human cancer growth. Oncogene.

[43] Guerra E., Trerotola M., Relli V., Lattanzio R., Ceci M., Boujnah K., Pantalone L., Di Pietro R., Iezzi M., Tinari N. (2023). The 2EF Antibody Targets a Unique N-Terminal Epitope of Trop-2 and Enhances the In Vivo Activity of the Cancer-Selective 2G10 Antibody. Cancers.

[44] Liu X., Luan L., Liu X., Jiang D., Deng J., Xu J., Yuan Y., Xing J., Chen B., Xing D. (2023). A novel nanobody-based HER2-targeting antibody exhibits potent synergistic antitumor efficacy in trastuzumab-resistant cancer cells. Front. Immunol..

[45] Verhaar E.R., Woodham A.W., Ploegh H.L. (2021). Nanobodies in cancer. Semin. Immunol..

[46] Hu Y., Wang Y., Lin J., Wu S., Lv H., Ji X., Wang S. (2022). Identification and Characterization of Specific Nanobodies against Trop-2 for Tumor Targeting. Int. J. Mol. Sci..

[47] Zhao D., Liu L., Liu X., Zhang J., Yin Y., Luan L., Jiang D., Yang X., Li L., Xiong H. (2022). A potent synthetic nanobody with broad-spectrum activity neutralizes SARS-CoV-2 virus and the Omicron variant BA.1 through a unique binding mode. J. Nanobiotechnol..

[48] Szala S., Froehlich M., Scollon M., Kasai Y., Steplewski Z., Koprowski H., Linnenbach A.J. (1990). Molecular cloning of cDNA for the carcinoma-associated antigen GA733-2. Proc. Natl. Acad. Sci. USA.

[49] Balzar M., Winter M., de Boer C., Litvinov S. (1999). The biology of the 17–1A antigen (Ep-CAM). J. Mol. Med..

[50] Stein R., Govindan S.V., Mattes M.J., Shih L.B., Griffiths G.L., Hansen H.J., Goldenberg D.M. (1999). Targeting human cancer xenografts with monoclonal antibodies labeled using radioiodinated, diethylenetriaminepentaacetic acid-appended peptides. Clin. Cancer Res..

[51] Stein R., Basu A., Chen S., Shih L.B., Goldenberg D.M. (1993). Specificity and properties of MAb RS7-3G11 and the antigen defined by this pancarcinoma monoclonal antibody. Int. J. Cancer.

[52] Govindan S.V., Stein R., Qu Z., Chen S., Andrews P., Ma H., Hansen H.J., Griffiths G.L., Horak I.D., Goldenberg D.M. (2004). Preclinical therapy of breast cancer with a radioiodinated humanized anti-EGP-1 monoclonal antibody: Advantage of a residualizing iodine radiolabel. Breast Cancer Res. Treat..

[53] Pavšič M. (2021). Trop2 Forms a Stable Dimer with Significant Structural Differences within the Membrane-Distal Region as Compared to EpCAM. Int. J. Mol. Sci..

[54] Abramson J., Adler J., Dunger J., Evans R., Green T., Pritzel A., Ronneberger O., Willmore L., Ballard A.J., Bambrick J. (2024). Accurate structure prediction of biomolecular interactions with AlphaFold 3. Nature.

[55] Upadhyay S.S., Balaya R.D.A., Parate S.S., Dagamajalu S., Prasad T.S.K., Shetty R., Raju R. (2023). An assembly of TROP2-mediated signaling events. J. Cell Commun. Signal..

[56] Abazari M.A., Soltani M., Kashkooli F.M. (2023). Targeted nano-sized drug delivery to heterogeneous solid tumor microvasculatures: Implications for immunoliposomes exhibiting bystander killing effect. Phys. Fluids.

[57] Nguyen T.D., Bordeau B.M., Balthasar J.P. (2023). Use of Payload Binding Selectivity Enhancers to Improve Therapeutic Index of Maytansinoid–Antibody–Drug Conjugates. Mol. Cancer Ther..

[58] Bordeau B.M., Nguyen T.D., Polli J.R., Chen P., Balthasar J.P. (2023). Payload-Binding Fab Fragments Increase the Therapeutic Index of MMAE Antibody-Drug Conjugates. Mol. Cancer Ther..

[59] van Rij C.M., Frielink C., Goldenberg D.M., Sharkey R.M., Lütje S., McBride W.J., Oyen W.J., Boerman O.C. (2014). Pretargeted Radioimmunotherapy of Prostate Cancer with an Anti-TROP-2×Anti-HSG Bispecific Antibody and a ^177^Lu-Labeled Peptide. Cancer Biother. Radiopharm..

[60] Chen W., Li M., Younis M.H., Barnhart T.E., Jiang D., Sun T., Lang J.M., Engle J.W., Zhou M., Cai W. (2022). ImmunoPET of trophoblast cell-surface antigen 2 (Trop-2) expression in pancreatic cancer. Eur. J. Nucl. Med..

[61] van Rij C.M., Frielink C., Goldenberg D.M., Sharkey R.M., Franssen G.M., Lütje S., McBride W.J., Oyen W.J.G., Boerman O.C. (2015). Pretargeted immunoPET of prostate cancer with an anti-TROP-2 x anti-HSG bispecific antibody in mice with PC3 xenografts. Mol. Imaging Biol..

[62] van Rij C.M., Lütje S., Frielink C., Sharkey R.M., Goldenberg D.M., Franssen G.M., McBride W.J., Rossi E.A., Oyen W.J.G., Boerman O.C. (2013). Pretargeted immuno-PET and radioimmunotherapy of prostate cancer with an anti-TROP-2 x anti-HSG bispecific antibody. Eur. J. Nucl. Med..

[63] Lütje S., Rijpkema M., Goldenberg D.M., van Rij C.M., Sharkey R.M., McBride W.J., Franssen G.M., Frielink C., Helfrich W., Oyen W.J. (2014). Pretargeted dual-modality immuno-SPECT and near-infrared fluorescence imaging for image-guided surgery of prostate cancer. Cancer Res..

[64] D’HUyvetter M., Vincke C., Xavier C., Aerts A., Impens N., Baatout S., De Raeve H., Muyldermans S., Caveliers V., Devoogdt N. (2014). Targeted radionuclide therapy with A 177Lu-labeled anti-HER2 nanobody. Theranostics.

[65] Gainkam L.O.T., Huang L., Caveliers V., Keyaerts M., Hernot S., Vaneycken I., Vanhove C., Revets H., De Baetselier P., Lahoutte T. (2008). Comparison of the biodistribution and tumor targeting of two 99mTc-labeled anti-EGFR nanobodies in mice, using pinhole SPECT/micro-CT. J. Nucl. Med..

