# Supplementary material for: Novel Anti-Trop2 Nanobodies Disrupt Receptor Dimerization and Inhibit Tumor Cell Growth
Source: Pharmaceutics. 2024 Sep 27;16(10):1255. doi: 10.3390/pharmaceutics16101255 (PMC11510716; doi:10.3390/pharmaceutics16101255)
Supplement: Supplementary file 1 [file pharmaceutics-16-01255-s001.zip › pharmaceutics-3094853-supplementary.pdf]

Novel anti-Trop2 nanobodies disrupt receptor dimerization and inhibit tumor cell growth

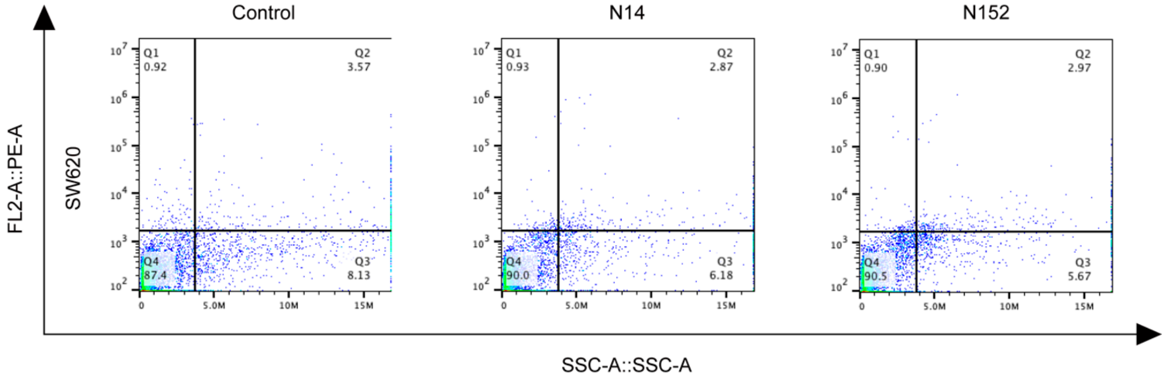

Figure S1. N14 and N152 do not bind to Trop2-low/negative tumor cells (SW620). Treatment of SW620 cells with either the control antibody (C5G2) or anti-Trop2 nanobodies (N14 and N152) resulted in nearly all cells remaining unstained and migrating to the lower left quadrant, amounting to 87.4%, 90.0%, and 90.5% for each group, respectively. These results demonstrates that N14 and N152 do not bind to Trop2-low/negative tumor cells, underscoring their specificity for Trop2.

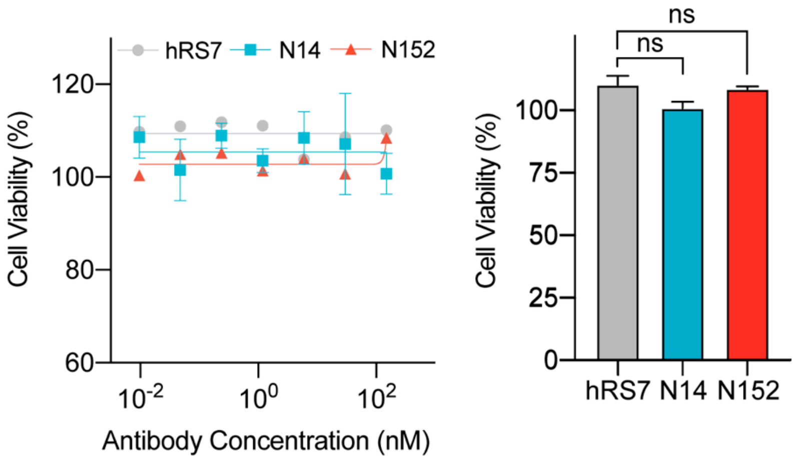

Figure S2. N14 and N152 do not affect the proliferation of Trop2-low/negative tumor cells (SW620). The data is presented through proliferation inhibition curves (Left) and the bar graphs (Right) at the maximum concentration. Regardless of whether treated with the control (hRS7) or anti Trop2 nanobodies (N14 and N152), there was no inhibition observed in the proliferation of SW620. Cell viability differences among groups were not statistically significant. The cell viability was reported as the mean ± SEM (n = 3). All statistical significance of the experiments was assessed using Student's *t*-test (ns, no significant; *p < 0.05; **p < 0.01; ***p < 0.001).
